# Current State of Pleural-Directed Adjuncts Against Malignant Pleural Mesothelioma

**DOI:** 10.3389/fonc.2022.886430

**Published:** 2022-05-02

**Authors:** Agnes Y. Choi, Anand Singh, Danyi Wang, Karthik Pittala, Chuong D. Hoang

**Affiliations:** Thoracic Surgery Branch, National Cancer Institute, National Institutes of Health, Bethesda, MD, United States

**Keywords:** malignant pleural mesothelioma, multimodality treatment, intraoperative adjuncts, intrathoracic, polymer, hydrogel, nanoparticle, microRNA

## Abstract

Multimodality therapy including surgical resection is the current paradigm in treating malignant pleural mesothelioma (MPM), a thoracic surface cancer without cure. The main limitation of all surgical approaches is the lack of long-term durability because macroscopic complete resection (R1 resection) commonly predisposes to locoregional relapse. Over the years, there have been many studies that describe various intrapleural strategies that aim to extend the effect of surgical resection. The majority of these approaches are intraoperative adjuvants. Broadly, there are three therapeutic classes that employ diverse agents. The most common, widely used group of adjuvants are comprised of direct therapeutics such as intracavitary chemotherapy (± hyperthermia). By comparison, the least commonly employed intrathoracic adjuvant is the class comprised of drug-device combinations like photodynamic therapy (PDT). But the most rapidly evolving (new) class with much potential for improved efficacy are therapeutics delivered by specialized drug vehicles such as a fibrin gel containing cisplatin. This review provides an updated perspective on pleural-directed adjuncts in the management of MPM as well as highlighting the most promising near-term technology breakthroughs.

## Introduction

**Figure 1:**
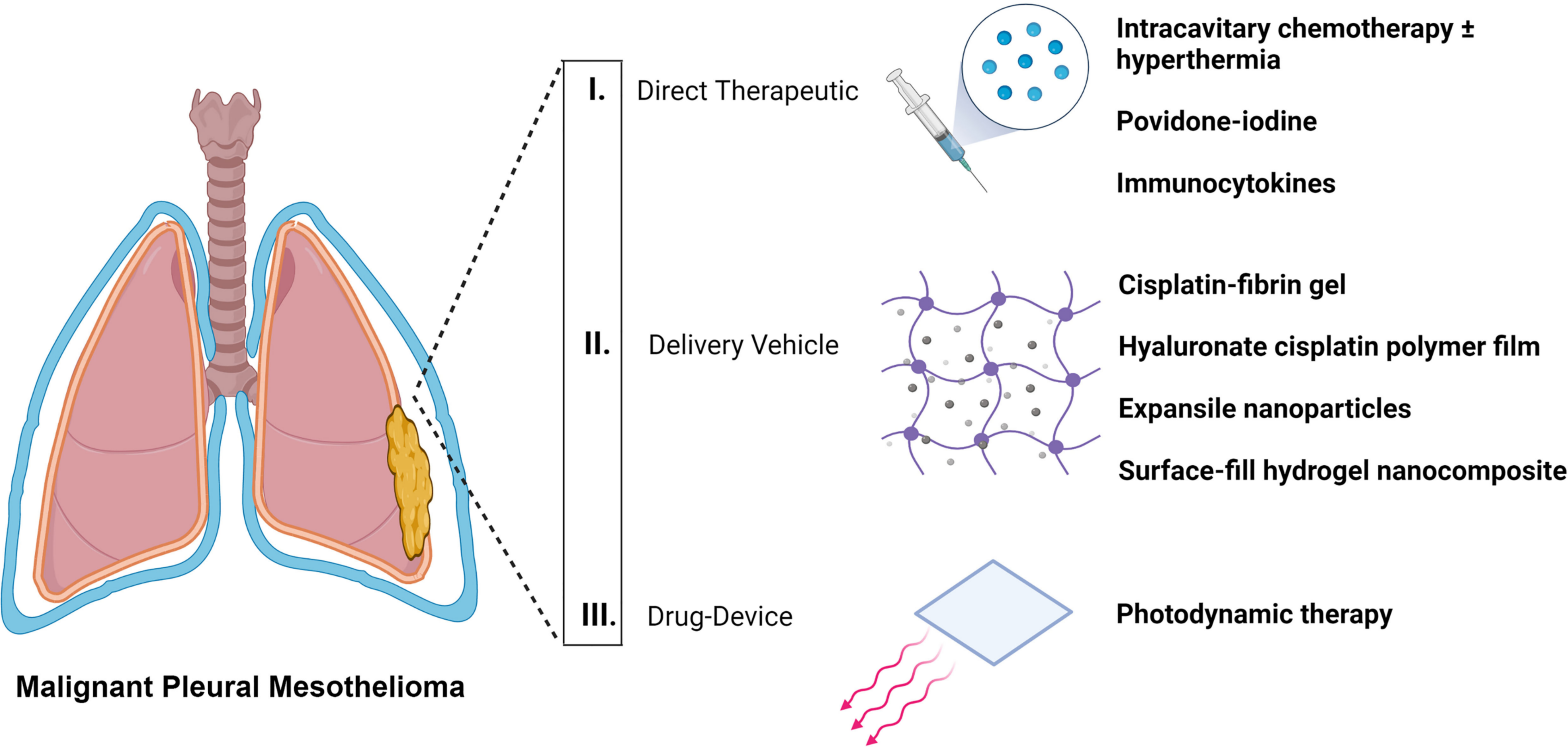
Classification of locoregional pleural-directed adjuncts for surgical-based therapy of mesothelioma.

Malignant pleural mesothelioma (MPM) is a rare, highly aggressive, and recalcitrant tumor arising from the mesothelial lining of the pleura. To date, there is no clinical standard (1) that might yield satisfactory long-term outcomes. It remains incurable. Selected patients, however, enjoy improved overall survival (OS) and recurrence-free survival with multimodality approaches. These strategies involve surgical resection by either extra-pleural pneumonectomy (EPP) or pleurectomy-decortication (PD) plus chemotherapy and/or radiotherapy (2, 3). The most critical success-limiting factor in the treatment of patients afflicted with MPM is the high recurrence rates of local disease ranging from 30% to 75% (4). This failure has prompted further investigations into intraoperative adjuncts to improve locoregional control by curbing microscopic residual foci (R1 margin) more effectively. In this review, we identify three major therapeutic classes (i.e., strategies) for pleural-directed adjuncts that summate the entire emerging field. We discuss the pros and cons of specific examples to illustrate the concept underlying each class of adjuvant that spans the application of direct therapeutics, to delivery vehicles carrying therapeutic(s), and to drug-device combinations (**Figure 1**, **Table 1**).

**Table 1:** Locoregional pleural-directed adjuncts against mesothelioma: pros & cons.

Pleural Adjuncts	Form Factor	Administration	Therapeutic Cargo	Cancer Targeting Mechanism	Toxicity	Current Status
**DIRECT THERAPEUTIC**						
Intracavitary chemotherapy ± hyperthermia (HIOC)	Liquid	Intracavitary perfusion	Chemotherapy	Unknown	Systemic effects Grade III+ AE	• Phase I/II studies • No standardized protocol
Povidone-iodine (PVP-I) ± hyperthermia	Liquid	Intracavitary perfusion	Povidone-iodine	Unknown	Systemic effects Grade I-III AE	• Single institution Phase I/II studies • No standardized protocol
Immunocytokines	Liquid	Intracavitary infusion	Interleukin-2	Activation of immune cells (LAK cells)	Systemic effects Grade I-II AE	• Single Phase II study • No standardized protocol • No follow-up studies
**DELIVERY VEHICLE**						
Cisplatin-fibrin gel	Gel	VATS spray-on application (Vivostat)	Chemotherapy	Unknown	Systemic effects Grade I-IV AE	• Single Phase I study • Lack of dose-dependent drug levels in tumor tissue • Phase II study started
Hyaluronate cisplatin (HYALCIS) film	Thin-Film	Direct surface application	Chemotherapy	Unknown	Systemic drug concentration No major effects	• Preclinical studies orthotopic xenograft tumor models • Preclinical large animal pharmacokinetic study • EMA & FDA approval
Expansile nanoparticles (eNP)	Nanoparticle	Intrapleural injection	Chemotherapy	Unknown	Not studied	• Preclinical study with orthotopic xenograft tumor models
Surface-fill hydrogel (SFH) nanocomposite	Hydrogel nanocomposite	Intrapleural injection and spray-on application	microRNA	Local application & Positive-charged microRNA-peptide nanoparticles selectively target negative-charged cancer cells	microRNA undetectable in systemic circulation No major effects	• Preclinical study with orthotopic xenograft tumor models
**DRUG-DEVICE**						
Photodynamic therapy (PDT)	Laser Light	Intracavitary + Systemic	Photosensitizer	Volume of light irradiation & uptake of photosensitizer & oxygen	Systemic effects (photosensitizer) Grade I-V AE	• Phase I-III studies • No standardized protocol • Unclear efficacy

AE, Adverse Events categorized according to common toxicity criteria (CTC) or Clavian-Dindo grade, EMA, European Medicines Agencyp; FDA, Food and Drug Administration (USA); VATS, Video-assisted Thoracoscopic Surgery.

## Direct Therapeutic

A focused review and update of intrapleural therapeutic modalities (i.e., agents) that can augment surgical resection and address microscopic residual tumor foci (R1 margin) is presented here. Particular attention is dedicated to verifying the molecular mechanisms of action for each type of agent used in this class of surgical adjuvants.

### Intracavitary Chemotherapy ± Hyperthermia

Results from intracavitary chemotherapy in ovarian carcinoma in the 1980s (5) led to pharmacokinetic studies of intrapleural cisplatin and mitomycin as intraoperative adjuvants to PD in the 1990s (6), followed by the first phase II trial in MPM patients (60-minute perfusion time without heating) (7). The use of intracavitary chemotherapy was predicated on some perceived advantages like improved drug penetration at higher doses to residual tumor foci and less systemic toxicity. Later, adding hyperthermia was thought to improve the efficacy of intracavitary chemotherapy by increasing absorption into cancer cells and potentiating the tumoricidal activity of the chemotherapy agent. A small study of 10 MPM patients compared PD with normothermic intracavitary cisplatin (100 mg/m^2^) versus PD or EPP with hyperthermic intraoperative chemotherapy (HIOC) at 41.5°C (8). A higher local tissue to perfusate ratio of cisplatin concentration after hyperthermic perfusion suggested a pharmacokinetic advantage imparted by heat. This observation, thus, paved the way for numerous HIOC studies in MPM patients.

Since 1994, there have been at least 20 studies using HIOC in MPM surgical studies (9). A recent phase I trial assessed safety and feasibility of combination drug HIOC in 59 patients undergoing EPP and 41 receiving PD (10). The observed morbidity rates in EPP and PD groups were 54% and 42%, respectively, while there were two perioperative deaths (2%). Dosing for cisplatin at 175 mg/m^2^ and gemcitabine at 1000 mg/m^2^ was established during 60 minutes of perfusion at 40 to 42°C. Median OS for patients with epithelioid histology was 26 and 59 months for the EPP versus PD cohorts, respectively, compared to 11 and 21 months for patients with non-epithelioid tumors. Similar outcomes were reported in a retrospective single institution study of 71 MPM patients who all underwent PD followed by 90 minutes of HIOC at 42°C using cisplatin (200 mg) and doxorubicin (100 mg) (11). For MPM HIOC studies to date (mostly observational, retrospective, underpowered phase I-II studies), cohorts ranged from four to 104 patients with survival between 11 and 36 months (9). Despite these encouraging outcomes with HIOC-based multimodality therapy, lack of improvement in locoregional control is still the major shortcoming. In a follow-up retrospective study of 132 patients undergoing EPP plus HIOC (cisplatin 175 – 225 mg/m^2^ for 60 minutes at 42°C) followed by adjuvant chemoradiation (according to modern techniques and standards), there was a disappointing overall recurrence rate of 75% (4). The ipsilateral hemithorax was the most common site of recurrence and both hemithoraces recurred independent of stage. Other non-thoracic sites of recurrence were observed more frequently according to higher stages. This study emphasizes the unreliable therapeutic effect of HIOC in MPM surgery and the critical need for better locoregional therapies.

Currently, there are no available phase III studies to help guide current multi-modality approaches for MPM. Meaningful comparisons of short- and long-term outcomes between HIOC regimens is impossible owing to diverse implementation of parameters like temperature, duration, type, numbers and combinations of chemotherapy agents, drug concentrations, volume of perfusate fluid, nephroprotective agents, etc. Thus, there is not yet a clinical consensus on a standardized HIOC procedure, although there is a multi-institution, international effort to define some basic working parameters (9). Aligned with this effort to realize the clinical value of HIOC, is the current call for acknowledging HIOC as a feasible practice to augment MPM surgical resection and that it should be mentioned as a therapeutic option in society guidelines, which will continue to evolve as more data becomes available in future trials (12).

Critical review of the literature reveals only a few, if any, basic science studies that lend to the fundamentals of HIOC mechanism of action. In fact, the precise mechanism of preferential drug delivery to cancer cells in the specific context of HIOC remains unknown (13). The bulk of experimental evidence purporting a rationale for using HIOC as a drug delivery strategy is based on older pharmacokinetic studies which inferred successful delivery/unloading of agent from concentration differences across tissue (chest wall or lungs) to perfusate content to the measured plasma levels (6, 8, 14). The relative contribution of temperature versus local drug dose to selectively kill cancer cells remains obscure (13). In one of the very few MPM-specific studies (*in vitro*) (15), the ongoing rationale to employ HIOC in multimodality therapy of MPM was called into question. When MPM cell lines were compared to other types of cancer cell lines, the MPM cells were not particularly heat sensitive and cisplatin alone was less effective. Importantly, the temperature of the perfusate did not consistently match the actual internal temperature of tissues/cells. Clinically relevant cancer cytotoxicity in this study did not occur until temperature exceeded 45°C, which is higher than temperatures commonly used in HIOC human trials (namely 42°C) (9).

### Povidone-Iodine

Povidone-iodine [PVP-I, poly-(1-vinyl-2-pyrrolidone)] is a time-honored antiseptic agent commonly used throughout clinical practices for handwashing, skin cleansing, and irrigation/lavage of body cavities for disinfection (16, 17). Since 2004, a single European group has been assessing the impact of adding hyperthermic PVP-I as an intraoperative adjuvant for MPM patients undergoing PD followed by prophylactic radiotherapy and later chemotherapy. The latest update on a cohort of 102 patients encompassing all MPM histologies and stages I-IV who received intraoperative PVP-I 10% (mixed with 5-6 L sterile water over 15 minutes total exposure time at 40 to 41°C) showed encouraging outcomes that continued to improve from their previous reports when the cohort size was 36 and 54 patients (18). The median OS was 32 months, and 5-year survival rate was 23.1%. Thirty-day mortality was nil and 30 patients (29.4%) sustained postoperative complications. Despite demonstrating feasibility and safety, further investigations of whether PVP-I use intrinsically impacts MPM outcomes and whether there is any necessity for hyperthermia are warranted.

The basic scientific support for this clinical practice is based on limited *in vitro* studies without any *in vivo* demonstration of PVP-I-specific effects in cancer cells. It is generally thought in human MPM cancer cell lines that PVP-I causes cellular necrosis *via* reactive oxygen intermediates which might contribute to stimulating anti-tumor inflammatory reactions (19, 20). One *in vitro* study showed that the necrotic phenotype (< 1% cell viability) was evident at 0.01 to 0.1% PVP-I concentration by 7.5 minutes post-exposure, without further improvement at longer exposures times up to 48 hours (20). Another *in vitro* study showed that sarcomatoid cells required a higher PVP-I concentration of 1% for cell killing while non-sarcomatoid histologies exhibited similar cell killing effect at the 0.1% concentration after a 10-minute incubation period (19). In both studies, no hyperthermia was needed in their experimental conditions. Moreover, cellular necrosis was a non-specific effect with similar cell killing observed in the MeT-5A “normal” pleural mesothelial cells (immortalized with SV40 large T antigen) (21). Importantly, no further experimental data were provided from animal studies or other *in vivo* results. Recently, there is conflicting data reported on the possible mechanism of cellular killing exerted by normothermic (37°C) PVP-I where thymic epithelial cells and MeT-5A were indiscriminately killed by cellular fixation after 30 minutes of exposure (22). Why different cells/tissues would die by distinctly different cellular mechanisms (cellular fixation is not necrosis or apoptotic cell death) using the identical PVP-I agent is perplexing.

### Immunocytokines

The least explored intrapleural adjuvant strategy to date is the direct use of immunomodulatory agents that can induce anti-tumor effects. A phase II trial of 49 stage II-III MPM patients explored the feasibility and efficacy of a unique four-modality intervention, including immunotherapy (23). Patients underwent: 1) pre-operative intrapleural IL-2 infusion *via* pigtail 12Fr catheter, 2) PD procedure, 3) post-operative sequential intrapleural chemotherapy followed with IL-2 infusions, and 4) adjuvant chemoradiotherapy plus long-term subcutaneous IL-2. Median OS reached 26 months without any operative fatality. Since then, there have not been more similar-minded studies in MPM.

Overall, this was a complicated clinical regimen. There was not an abundance of convincing preliminary data to support all elements of trial design, much less in combining them all. Consequently, it has been difficult to discern why this trial was conceived as such since there was not an easily recognized logical step-building from previous trials. In fact, there was not a specific hypothesis stated. The total length of treatment for enrolled patients was never specified (at least 4 months from study entry to the start of maintenance immunotherapy). The final outcomes were difficult to discern, especially in being able to pinpoint whether surgery, immunotherapy, chemotherapy, or radiation (and in what order: pre-, intra-, post-operative) was most beneficial. The role of immunotherapy as an intrapleural adjuvant remains undefined, yet should be revisited now in the context of approved frontline use of immune checkpoint inhibitors in certain scenarios of MPM (24).

Pre-clinical mechanistic studies directly warranting use of intrapleural IL-2 therapy in MPM treatment are sparse. An *in vitro* study demonstrated that IL-2 in combination with lymphokine-activated killer cells could effectively lyse the MPM cells whereas NK cells were ineffective (25, 26). Extrapolation of *in vitro* results and success of cytokine therapy in other solid tumors ultimately led to, for example, a phase II MPM trial of intrapleural IL-2 as frontline monotherapy (n = 22 patients) (27). Over one-half (55%) of the patients showed at least a partial response with median OS of 18 months, but this was complicated by significant systemic toxicities. Subsequently, widespread adoption of immunocytokines as part of a multimodal strategy never fully materialized. The pharmacokinetics of (28) and the detailed cellular mechanisms and all relevant cell effectors that are induced by intrapleural IL-2 remain incompletely characterized *in vivo* in the context of MPM.

## Delivery Vehicle Carrying Therapeutic

Newer strategies for the delivery of intracavitary local therapy are being developed and tested in pre-clinical models and human trials. The broad aim is to achieve durable and effective treatments against MPM by leveraging novel technologies to specifically enhance cancer cell-targeted strategies. These drug delivery depot systems (i.e., vehicle) encompass a variety of form-factors such as drug-releasing gels, thin films, or nanoparticles. It is anticipated that when these approaches mature, improved clinical efficacy over intrapleural application of direct therapeutics may be attained.

### Cisplatin-Fibrin Gel

Building upon the clinical experience of pleural-directed drug adjuvants to improve the local effect of MPM surgical resection, therapeutic agents have been combined with delivery vehicles to maximize local drug concentrations while limiting systemic adverse events. Pre-clinical MPM studies demonstrated anti-tumor efficacy using cisplatin combined with fibrin (gel) delivered by an intracavitary injection technique (29, 30). The intracavitary cisplatin-fibrin treatment increased local cisplatin tissue concentrations while significantly reducing systemic cisplatin distribution as compared to the chemotherapeutic solution alone.

A phase I dose escalation trial followed, in which 12 non-sarcomatoid MPM patients with mostly stages III-IV underwent extended PD procedure (31). Cisplatin was mixed with patient autologous plasma-derived fibrin which was prepared using the Vivostat system (32) where the cisplatin-fibrin gel was sprayed on pleural surfaces intraoperatively. The mortality rate at 30 and 90 days was 0% and four patients (33%) experienced major morbidity. Median OS was 21 months with a median freedom from recurrence of 8 months, where 83% of patients recurred at 1-year post-operative. Based on tissues biopsies of the chest wall, cytotoxic cisplatin concentrations (12-133 μg/g) were achieved for all treatment dose levels. Moreover, drug levels remained detectable in chest wall musculature for extended timepoints (cytotoxic concentrations in one patient at day 74 and detectable non-cytotoxic levels in another patient beyond 6 months of therapy). Despite locoregional administration, cisplatin distributed systemically and 10% to 27% of the total cisplatin was excreted in the urine within the first 48 hours. Tissue cisplatin levels were highly variable, not dose dependent. Overall, this study demonstrated safety and feasibility of cisplatin-fibrin gel, leading to initiation of a phase II trial.

However, many questions remain for further investigation. How the fibrin interacts with cisplatin and specifically how it forms a conjugate with cisplatin remains unknown because no mechanistic data have been presented. Whether the fibrin has any impact on the efficacy of cisplatin is unknown. Most importantly, the mechanism of cisplatin release from fibrin still needs to be investigated. These unknowns contribute to the lack of dose-dependent cisplatin tissue levels and its high variability as measured in each patient (i.e., inconsistent dose delivery). Without this prerequisite knowledge, it remains uncertain whether this delivery strategy will be able to selectively target tumor cells/tissues and, thereby, be any more efficacious compared to direct instillation of cisplatin. The off-target effects of cisplatin-fibrin are concerning as reflected by the urine excretion of systemic-leak cisplatin and its persistence in deep tissues outside of the pleural surfaces where MPM originates.

### Hyaluronate Cisplatin Polymer Film

Hyaluronate (hyaluronan or hyaluronic acid) is a polysaccharide of repeating units of D-glucuronic acid and (1-β-3) N-acetyl-D-glucosamine with physiochemical attributes useful in drug delivery strategies (33). Polymer flexible thin sheets of hyaluronate loaded with cisplatin (HYALCIS) have thus been applied in an orthotopic rat MPM recurrence model (after pneumonectomy) to investigate efficacy and toxicity as compared to direct cisplatin solution (34). Oddly, the cisplatin level in rat pleural tissue at autopsy was lower in the HYALCIS group compared to intrapleural cisplatin. Compared to direct intrapleural cisplatin, significant cisplatin levels (6- to 7-fold higher) were detected in plasma over an extended time (6 days). Histologic and biochemistry tests did not reveal major systemic toxicity in HYALCIS-treated mice. A follow-up pharmacokinetic study in an ovine (non-tumor) pneumonectomy model treated with 1% w/w HYALCIS films demonstrated the feasibility of inserting large polymer sheets into an animal cavity and delivery of relevant doses of cisplatin *in vivo* (35). Cisplatin concentrations in diaphragm, parietal pleura, and pericardium were markedly higher than those of intrapleural cisplatin solution and intravenous cisplatin for up to 24 days. However, cisplatin levels increased dramatically in plasma after treatments and continued to persist at clinically relevant levels for over 21 days despite there being no reported major systemic toxicities in treatment subjects.

These pre-clinical results support the next step of human trials that have yet to be conducted. Polymer films loaded with cisplatin for intrapleural therapy have been approved by regulatory agencies in Europe and the U.S. (e.g., Food and Drug Administration). Nevertheless, many facets of this technology remain obscure and could potentially predict suboptimal outcomes in future clinical settings. This methodology lacks a cancer-specific targeting mechanism. None of the *in vivo* studies in MPM have directly traced the fate of cisplatin once released from the hyaluronate film. Despite no major systemic side-effects in animals, there remains concern for treatment-related morbidity in humans considering the significant higher plasma levels seen with HYALCIS. More basic research is needed to understand the impact of the hyaluronate-cisplatin complex (36) to improve release kinetics and drug elimination rates. Furthermore, it remains unclear how efficiently a polymer sheet would perform in non-pneumonectomy situations which represent a more physically challenging anatomic landscape to effectively coat. Uneven application in areas where sufficient contact with certain surfaces is more difficult to achieve (e.g., curved lung lobes) may result in inconsistent drug delivery. Additionally, the possibility of even higher systemic drug absorption and secondary toxicity with the application of HYALCIS to the lung remains unknown.

### Expansile Nanoparticles

An alternative strategy to locoregional drug delivery can be achieved with novel synthetic polymer nanoparticle carriers (100 nm diameter) which swell upon exposure to acidic pH and subsequently release their therapeutic cargo within 24 hours (i.e., expansile nanoparticles, eNP) (37). A study using murine MPM orthotopic xenograft models demonstrated that macroscopic complete resection of tumor vis-à-vis pneumonectomy followed by intrapleural multidose (3x) treatment with paclitaxel-loaded eNP more than doubled median OS (55 vs. 22 days) as compared to controls (38). Ultraviolet light showed that intrapleural injection of fluorescent-labeled paclitaxel-loaded eNP co-localized to unresected tumor deposits (4 days post-treatment) and immunohistology showed that the nanoparticle construct further found its way into cancer cells. Thus, locoregional nanoparticle drug delivery was feasible post-resection and represents another potential strategy in multimodality MPM treatment.

This study did not explain, however, why the paclitaxel-loaded eNP remained intact once inside cancer cells in the co-localization studies (both *in vitro* and *in vivo*). There were no direct data to indicate the drug was properly unloaded intracellularly, nor were there supporting results measuring tissue levels of drug after injection of eNP. It will be interesting to see this technology mature with the flexibility to load other drugs that are known to have better intrinsic efficacy against MPM. More research is needed to elucidate the mechanism of eNP drug binding and the mechanism/kinetics of drug release as it remains unknown at this time. Future studies will need to distinguish the cytotoxic effect of eNPs from the effects of physical cell swelling, in addition to how eNPs preferentially target cancer cells *in vivo*. Furthermore, the fate of how eNPs are metabolized *in vivo* will have to be traced to assess for any off-target systemic toxic side-effects.

### Surface-Fill Hydrogel Nanocomposite

Recently, a new materials platform harnessing the compelling therapeutic traits of microRNA (39) (miRNA or miR) that can resolve some of the limitations of MPM intracavitary therapies has been described. A novel biodegradable thin-film depot and delivery material consisting of nanoparticles, prepared by complexing amphiphilic cationic peptides (first polymer) with negatively charged miRNA, which are then embedded into a shear-thinning, self-assembling peptide (second polymer) hydrogel, exerted preferential anti-cancer effects in several murine MPM xenografts (40). This peptide-based surface-fill hydrogel (SFH) nanocomposite can be applied directly to a body cavity *via* percutaneous or surgical access by syringe injection or sprayed to coat anatomic surfaces. After application, positively charged peptide-miRNA spherical nanoparticles (~150 nm diameter) are released over time from the net-positive charged hydrogel matrix to adjacent tissues and taken up more selectively by cancer cells [net-negative surface charge (41)]. The particle surface charge state, its size, miRNA encapsulation efficiency, and the peptide’s conformation are important for clathrin-mediated cell entry and endosomal trafficking. Once internalized, miRNA is released from the peptide nanoparticle and capable of affecting cellular function.

Biodistribution analysis of different anatomic sites after intrapleural treatment with miRNA-loaded SFH revealed that the miRNA preferentially reached MPM cells without observable delivery to other vital organs. The ability of SFH to deliver its payload locally was further confirmed by the lack of detectable miRNA in circulating plasma over a series of timed experiments. A systematic histopathologic and biochemical analysis in the murine models revealed no significant toxicities. Furthermore, the efficacy of SFH with encapsulated miR-215 (42) or miR-206 (43) nanoparticles in a single administration were evaluated in pre-clinical models of MPM. Tumor resection sites receiving adjuvant SFH miR-215 or miR-206 therapy showed minimal tumor recurrence compared to resection controls without miRNA treatment (40).

Unlike the other modalities, this SFH delivery depot has a precisely described mechanism for selective cancer cell killing which is dependent on the local delivery route, biophysical properties of the hydrogel, the deranged miRNA profile of the tumor, and relative resilience of normal tissues to miRNA perturbation (42). As this technology continues to mature, it will be interesting to see if efficacy and/or durability can be improved with augmentation such as using a cocktail of anti-MPM miRNA or novel drug and miRNA combinations. Potential drawbacks could be the relative high cost of such biomaterials or the lack of pre-existing infrastructure for large-scale manufacturing. Nevertheless, novel biomaterials with cancer-selective effects represent a promising MPM treatment strategy.

## Drug-Device Combination

In contrast to single modality approaches, drug-device combinations are inherently more complex owing to requirements of drug/agent design in tandem with technology and manufacturing innovations of the accompanying device hardware.

### Photodynamic Therapy

Photodynamic therapy (PDT) is a unique approach to treat MPM that relies on wavelength-specific visible light generated by a laser device focused on target tissues which have accumulated light-absorbing photosensitizing agent (i.e., drug) in the presence of oxygen. The localized interaction of these three components induces a tumoricidal photochemical reaction to produce reactive singlet oxygen leading to damage of the tumor cell wall and neovasculature (44). After MPM resection, light detectors are placed intracavitary to monitor light dose, light is delivered by a hand-held laser fiber device that illuminates surfaces where it is pointed towards while the chest cavity is filled with light-dispersing intralipid solution (45). Multiple factors can be adjusted to produce a desired cell killing effect customizable to the clinical situation and anatomy of specific patients including photosensitizer agent and dose, target tissue geometry, mode of light application, light source, irradiation parameters (e.g., wavelength), interval between drug and light illumination, etc. PDT can be administered repeatedly without cumulative toxicity and does not hinder other therapies or could be synergistic with certain specialized modalities. Depth of penetration is limited by wavelength and typically 5 to 10 mm of therapeutic effect can be achieved in clinical scenarios (46).

Since 1991, there have been 11 feasibility and safety trials, three retrospective survival studies and two prospective trials totaling 337 MPM patients who received intrapleural PDT during multimodality therapy of MPM including macroscopic complete resection (47). Since the trials were so heterogeneous any firm conclusions about PDT and its specific effect on OS are unrealistic. The lone phase III trial assessing PDT in MPM patients with maximal debulking surgery found no difference in median OS in PDT (14.4 months, n = 25 patients) versus no-PDT (14.1 months, n = 23 patients), nor in disease-free survival (8.5 months versus 7.7 months, respectively) (48). In contrast, a non-randomized prospective study comparing 14 PD plus PDT with hyperbaric oxygen patients versus 11 PD no-PDT patients, demonstrated significantly improved median OS (15 versus 10 months, respectively) and recurrence incidence of 4/14 versus 8/11, respectively (49). Neither study included enough patients to reach statistical power. Use of a hyperbaric oxygenation chamber is logistically self-limiting and unlikely to be widely adopted in hospitals. There is, nevertheless, good demonstration of PDT safety and feasibility by different institutions as well as tolerability with overall low toxicity in modern regimens. More clinical insight on the role for PDT is awaited as there is an ongoing randomized phase II trial of radical PD with or without intraoperative PDT (NCT02153229).

With apparent lack of superior efficacy over other local ablation strategies, more basic investigations are necessary to improve upon inherent shortcomings of this technology in the context of cancer surgery. The chest cavity geometry is a very challenging location to ensure even and consistent irradiation of light, especially when the intralipid solution cannot entirely fill up the chest during surgical procedures. More innovation is needed in devices to deliver PDT. Anticancer effects are dependent on oxygen levels in close proximity to tumor tissue, but the MPM microenvironment is characteristically hypoxic (50). Even more challenging is how to ensure co-localization of the photosensitizer agent along with molecular oxygen in critical subcellular organelles (e.g., mitochondria) to take full advantage of the cytotoxic effects of singlet oxygen. Most importantly, there is need for preferential cancer cell-specific targeting of systemically administered photosensitizer. The photosensitizers currently in use assume there is an enhanced permeability and retention effect that occurs in tumors, but this phenomenon has been called into question (51).

## Conclusions

MPM remains a fatal disease in need of highly effective therapeutic agents and strategies. Macroscopic complete resection as part of multimodality care certainly can contribute to positive outcomes. However, verifying a direct effect and quantifying the relative merit of surgical resection when used in a multimodality treatment scheme has been elusive. The lack of good clinical outcomes in MPM therapy is impacted by numerous heterogeneous factors including, for example: extent of surgical resection (EPP vs. PD), tumor histology (e.g., epithelioid vs. sarcomatoid), systemic therapy (neoadjuvant vs. adjuvant, etc.), and how the interventions are employed in a sequence. Such clinical parameters add complexity to the process of study designs best equipped to demonstrate direct benefits of pleural-directed adjuncts in MPM therapy (**Table 2**). Yet, an emerging trend is that pleural-directed adjuncts for control of microscopic residual disease (R1 margin) are a promising group of approaches that merit further investigation and development. Despite direct instillation of chemotherapy drugs being the most widely used, data from large, randomized trials are not available to guide clinical practices according to any standardized technique. It is likely that other novel drug-device combinations may be described in the near future that can address some of the major limitations of PDT, which has been largely underutilized. The most promising class of adjuncts dependent on a vehicle carrier to deliver local therapeutics is likely to receive more attention as biomedical technologies improve.

**Table 2:** Diverse mesothelioma treatment studies with a component of Intrapleural therapy.

Author	Cohort Size (N)	Study Design	Surgery	Epithelioid Histology	Neoadjuvant Drug Therapy	Pleural Therapy		Timing	Adjuvant Therapy
**DIRECT THERAPEUTIC**									
Rusch et al. (7)	27	Phase II	PD	70% (19/27)	None	Cisplatin + mitomycin		Intraoperative	Cisplatin + mitomycin
Ratto et al. (8)	10	Phase I	PD (6/10) EPP (4/10)	Not specified	None	PD - Cisplatin (3/10) - Heated cisplatin (3/10)	EPP - Heated cisplatin (4/10)	Intraoperative	Radiotherapy
Burt et al. (10)	104	Phase I	PD (41/104) EPP (59/104) Debulk (4/104)	PD: 71% (29/41) EPP: 53% (31/59) Debulk: 50% (2/4)	None	Heated cisplatin + gemcitabine		Intraoperative	Discretionary chemotherapy radiotherapy
Klotz et al. (11)	71	Retrospective cohort	Extended PD	77% (55/71)	Cisplatin + navelbine or pemetrexed	Heated cisplatin + doxorubicin		Intraoperative	None
Lang-Lazdunski et al. (17)	102	Phase I/II	PD	72% (73/102)	Cisplatin + pemetrexed	Heated Povidone Iodine		Intraoperative	Radiotherapy Cisplatin ± pemetrexed
Lucchi et al. (22)	49	Phase II	PD	80% (39/49)	Intrapleural Interleukin-2	Epidoxorubicin + Interleukin-2		Postoperative	†Cisplatin + gemcitabine + Interleukin-2
**DELIVERY VEHICLE**									
Opitz et al. (30)	12	Phase I	Extended PD	67% (8/12)	Cisplatin + pemetrexed	Cisplatin-fibrin gel		Intraoperative	None
**DRUG-DEVICE**									
Pass et al. (47)	48	Phase III	PD (23/48) EPP (25/48)	69% (33/48)	None	Photodynamic therapy		Intraoperative	Cisplatin + interferon-α 2b + tamoxifen
Matzi et al. (48)	34	Phase II	PD	62% (21/34)	None	Photodynamic therapy Hyperbaric oxygen		Intraoperative	None

PD, Pleurectomy-decortication; EPP, extra-pleural pneumonectomy, †Postoperative radiation was administered prior to adjuvant chemo-immunotherapy.

## Author Contributions

AC, AS, DW, and KP - writing of text and table, drawing figure, and editing. CH - writing, revision and editing, concept and design. All authors contributed to the article and approved the submitted version.

## Funding

This work was supported by NIH Intramural Research Program with funding from National Cancer Institute - Center for Cancer Research Grant ZIA BC 011657 (to CH).

## Conflict of Interest

The authors declare that the research was conducted in the absence of any commercial or financial relationships that could be construed as a potential conflict of interest.

## Publisher’s Note

All claims expressed in this article are solely those of the authors and do not necessarily represent those of their affiliated organizations, or those of the publisher, the editors and the reviewers. Any product that may be evaluated in this article, or claim that may be made by its manufacturer, is not guaranteed or endorsed by the publisher.
